# Host Soluble Factors Cause Changes in *Staphylococcus epidermidis* Antibiotic Susceptibility and Biofilm Formation Ability

**DOI:** 10.3390/pathogens12081064

**Published:** 2023-08-19

**Authors:** Fernando Oliveira, Vânia Gaio, Susana Brás, Sofia Oliveira, Angela França

**Affiliations:** 1 Centre of Biological Engineering, LIBRO—Laboratory of Research in Biofilms Rosário Oliveira, University of Minho, Campus de Gualtar, 4710-057 Braga, Portugal; 2 LABBELS—Associate Laboratory, 4710-057 Guimarães/Braga, Portugal

**Keywords:** staphylococcal infections, serum, plasma, anti-bacterial agents, biofilms, gene expression

## Abstract

*Staphylococcus epidermidis* is a major nosocomial pathogen with a remarkable ability to adhere to the surfaces of indwelling medical devices and form biofilms. Unlike other nosocomial pathogens, the interaction of *S. epidermidis* with host factors has not been the focus of substantial research. This study aimed to assess the alterations in the antibiotic susceptibility and biofilm formation ability of *S. epidermidis* in the presence of host serum factors. *S. epidermidis* strain RP62A was cultured in a laboratory culture medium with or without human serum/plasma, and changes in antibiotic susceptibility, biofilm formation, and gene expression were evaluated. The data obtained revealed that exposure to host serum factors increased the susceptibility of *S. epidermidis* to glycopeptide antibiotics and was also detrimental to biofilm formation. Gene expression analysis revealed downregulation of both *dltA* and *fmtC* genes shortly after human serum/plasma exposure. The importance of transferrin-mediated iron sequestration as a host anti-biofilm strategy against *S. epidermidis* was also emphasized. We have demonstrated that serum factors play a pivotal role as part of the host’s anti-infective strategy against *S. epidermidis* infections, highlighting the importance of incorporating such factors during in vitro studies with this pathogen.

## 1. Introduction

The increasing use of indwelling medical devices (e.g., catheters and prosthetic joints) is one of the major advancements in modern medicine, but it has also come at the price of being a major cause of healthcare-associated infections [1]. *Staphylococcus epidermidis*, a predominant species in the human skin microbiota, is a frequent cause of bloodstream infections and sepsis as it can adhere to and form biofilms on vascular catheters [2]. Even though most infections remain treatable with currently available antibiotic therapy, the global dissemination of multidrug-resistant lineages of *S. epidermidis* exhibiting reduced susceptibility to last-line antibiotics, such as vancomycin and teicoplanin [3], is a matter of concern.

While our knowledge of the pathogenic mechanisms of *S. epidermidis* has increased considerably, there is still a lack of in vitro studies performed under experimental conditions that properly reproduce the conditions that this pathogen experiences during infection. To better understand *S. epidermidis* pathogenesis, it is important to consider the actual role of host factors in the expression of virulence traits, such as biofilm formation. Previously, we have demonstrated that blood acts as an important external cue that triggers significant alterations in the transcriptome of *S. epidermidis* biofilm cells [4] and that plasma alone is responsible for significant transcriptional changes of genes involved in biofilm formation and immune evasion [5,6]. Specific binding of bacteria to host proteins found in the blood has long been regarded as a major facilitator of bacterial adhesion to medical devices, which stems from the fact that their surface becomes coated with host proteins shortly after insertion into the body [7]. Although *S. epidermidis* is known to express a wide range of molecules that bind to host proteins, such as fibronectin, fibrinogen or vitronectin, the specificity of this binding is still debatable [8]. Additionally, the presence of human serum has been shown to inhibit biofilm formation on artificial surfaces by staphylococci [9,10], casting doubts about the actual role of serum proteins in bacterial adhesion and biofilm formation in these bacteria. The contribution of host factors is even more relevant at the level of antibiotic susceptibility studies, as the reversible binding of antibiotics to blood proteins, commonly referred to as plasma protein binding, may affect their antimicrobial activity [11]. Another mechanism worth exploring is the potential of host factors to induce changes in the bacterial surface charge. Some studies on *S. aureus* have demonstrated that such alterations can impact its susceptibility to certain antibiotics [12,13]. Therefore, the use of the host’s biological fluids, such as human serum or plasma, has been considered to provide a more accurate prediction of antibiotic efficacy in vivo [11,14]. However, and to the best of our knowledge, there are no available studies thoroughly assessing the effect of human serum factors on the activity of different antibiotics against *S. epidermidis*.

Hence, this study aimed to assess if the exposure of *S. epidermidis* to environmental signals found during bloodstream infections, provided by human serum or plasma, induces significant alterations in its susceptibility to antibiotics and its biofilm formation ability.

## 2. Materials and Methods

### 2.1. Bacterial Strains and Chemicals

*S. epidermidis* ATCC 35984 (RP62A) was used in this study. For each experiment, isolated colonies were picked from tryptic soy agar (TSA, Liofilchem, Téramo, Italy) plates, inoculated into tryptic soy broth (TSB) (Liofilchem) and incubated overnight (~16 h) at 37 °C and 120 rpm (ES-20 Shaker-Incubator, BioSan, Riga, Latvia). Dicloxacillin, rifampicin, and teicoplanin were purchased from Sigma-Aldrich (St. Louis, MO, USA), tetracycline was purchased from GRiSP (Porto, Portugal), and vancomycin was purchased from PanReac Applichem (Darmstadt, Germany). Pooled human serum from clotted whole blood was purchased from Sigma-Aldrich, and pooled human plasma in lithium heparin was purchased from Tebu-Bio (Le Perray-en-Yvelines, France). TSB supplemented with lithium heparin (TSBH, BD Vacutainer, Franklin Lakes, NJ, USA) was used as a control in every experiment. Heat inactivation of serum/plasma was carried out at 56 °C for 30 min. Iron (III) chloride and human transferrin (partially iron-saturated) were purchased from Sigma-Aldrich.

### 2.2. Antibiotic Susceptibility Assays

Minimum inhibitory and bactericidal concentrations (MIC and MBC, respectively) were determined according to the Clinical Laboratory Standards Institute [15], using planktonic cells and TSB or serum/plasma-enriched TSB as the growth medium. For quality control purposes, *Staphylococcus aureus* ATCC 29213 was used in all experiments. For experiments using antibiotics at their peak serum concentration (PSC), overnight-grown bacteria were diluted in TSB or serum/plasma-enriched (10–30%) TSB to an optical density, at 620 nm (OD_620_), ~0.1 (~7 × 10^7^ CFU/mL), and then incubated at 37 °C and 120 rpm for 4 h to reach the exponential phase. The samples were then sonicated for 10 s at 30% amplitude (Cole-Parmer 750-W Ultrasonic Homogenizer 230 VAC, Cole-Palmer, Vernon Hills, IL, USA) to disrupt cell clusters and the bacterial suspensions diluted in TSB or serum/plasma-enriched (10–30%) TSB to a final concentration of ~2 × 10^7^ CFU/mL. Of note, the sonication cycle used did not compromise cell viability as previously determined [16]. Afterwards, each antibiotic was added to the previous suspensions at the respective PSC, and the cultures were incubated at 37 °C and 120 rpm for 3 h. Control suspensions without antibiotics were also included. After 3 h of incubation with antibiotics, 1 mL of each suspension was collected and centrifuged at 4 °C and 16,000× *g* for 10 min, and the pellets were suspended in 1 mL of 0.9% NaCl. The samples were then sonicated as above before by performing CFU counts.

### 2.3. Biofilm Formation Assays

Biofilms were grown on flat bottom 96-well polystyrene plastic microplates (Orange Scientific, Braine-l’Alleud, Belgium). An overnight culture was adjusted, in TSB, to an OD_620_ of ~0.25, which is equivalent to ~2 × 10^8^ CFU/mL, and 2 µL of this suspension was used to inoculate 198 µL of TSB supplemented with 0.4% (*w*/*v*) glucose (Thermo Fisher Scientific Inc., Waltham, MA, USA) (TSBG) or serum/plasma-enriched (10–30%) TSBG. The microplates were incubated at 37 °C and 120 rpm for 24 h. For the precoating experiments, 200 µL of plasma or serum (100%) was added to a microplate and incubated overnight at 37 °C. Afterwards, the solutions were removed, and the biofilms were grown as described above. For the experiments with preformed 24 h-old biofilms, the biofilms were grown as mentioned before. Then, the culture supernatants were removed carefully, the biofilms were washed twice with 200 µL of 0.9% NaCl, and 200 µL of serum/plasma-enriched (10–50%) TSBG solution was added. The microplates were incubated for an additional 24 h, as detailed above. For quantification of biofilm biomass, the culture supernatants were removed and the biofilms were washed twice with 200 µL of 0.9% NaCl and then stained with crystal violet, as optimized before [17]. Briefly, the biofilms were fixed with methanol (Thermo Fisher Scientific Inc.) for 20 min and stained with 1% (*v*/*v*) crystal violet (Merck Millipore, Darmstadt, Germany) for 15 min. The excess stain was rinsed off with tap water, and the stain bound to the biofilm was solubilized with 33% (*v*/*v*) glacial acetic acid (Thermo Fisher Scientific Inc.). Absorbance was measured at 595 nm (A_595nm_) using a microplate reader (Biochrom EZ Read 800 Plus, Biochrom, Cambridge, UK).

### 2.4. Planktonic Growth Curves

Overnight-grown bacteria were diluted in TSB or serum/plasma-enriched (10–50%) TSB to an OD_620nm_ ~0.1 (~7 × 10^7^ CFU/mL) and then incubated at 37 °C and 120 rpm. At the time points indicated, aliquots were collected for OD_620nm_ measurements and CFU counting. Before any measurement, the samples were sonicated as mentioned before.

### 2.5. Gene Expression Quantification by qPCR

RNA extraction from the *S. epidermidis* cultures grown in either TSB/TSBH or serum/plasma-enriched (20%) TSB (for 45 or 90 min) was performed using the E.Z.N.A.^®^ Total RNA Kit I (Omega Bio-Tek, Norcross, GA, USA), with some previously optimized modifications [18]. Genomic DNA was degraded by using DNase I (Thermo Fisher Scientific Inc.), and RNA quantity and purity were assessed using a NanoDrop One (Thermo Fisher Scientific Inc.). RNA integrity was assessed by non-denaturing electrophoresis [19]. cDNA was diluted 1:200 and tested in quantitative PCR (qPCR) using the primer pairs depicted in Table S1 and following Xpert Fast SYBR Mastermix (GRiSP, Porto, Portugal) instructions. qPCR runs were performed in a CFX96 (Bio-Rad, Hercules, CA, USA) with the following cycle parameters: 95 °C for 3 min and 40 cycles of 95 °C for 5 s and 60 °C for 25 s. Melt analysis was performed to ensure the absence of unspecific products and primer-dimers. The expression of the genes tested was normalized to the expression of the reference genes *16S rRNA* and *gyrB* using the Pfaffl method [20] and considering TSB (in the case of serum) or TSBH (in the case of plasma) as the control conditions. The primers were designed with Primer3 [21] using the *S. epidermidis* RP62A genome sequence (NCBI accession no. NC_002976.3) as a template. mFold was used for the prediction of secondary structures [22], and primer specificity was confirmed using Primer-BLAST [23]. PCR amplification efficiency was determined from the slope of a standard curve.

### 2.6. Zeta Potential Measurements

Overnight-grown bacteria were diluted in TSB or serum-enriched (30%) TSB to an OD620 ~0.1 (~7 × 10^7^ CFU/mL) and then incubated at 37 °C and 120 rpm for 4 h. After incubation, the bacterial cells were washed twice with 0.5 mM potassium phosphate buffer (pH 7.4) and then sonicated as previously described. A bacterial suspension containing ~2 × 10^8^ CFU/mL was prepared in 0.5 mM potassium phosphate buffer, and zeta potentials were determined using a Malvern Zetasizer Nano ZS (Malvern Panalytical, Malvern, UK).

### 2.7. Statistical Analysis

Statistical significance was determined using GraphPad Prism version 7.0a. The statistical tests used, significance values, and group sizes are described in the figure legends. Significance was defined as *p* < 0.05.

## 3. Results

### 3.1. Effect of Host Serum Factors on Susceptibility to Antibiotics

Despite its importance as a nosocomial pathogen, little is known about the role of host serum factors in the susceptibility of *S. epidermidis* towards antibiotic treatment. In this study, we assessed the activity of selected antibiotics in media supplemented with increasing concentrations of serum or plasma. Our selection covered different classes of antibiotics, with different mechanisms of action, which are commonly employed in the treatment of staphylococcal infections (Table S2). We first determined the MIC and MBC of each antibiotic using the standard broth microdilution method (Tables S3 and S4). While the MICs of tetracycline and vancomycin remained unchanged in the presence of human serum or plasma (0.25 mg/L and 2 mg/L, respectively), the MIC of rifampicin was increased by a factor of up to 8 (from 0.004 to 0.031 mg/L). For teicoplanin, it was rather difficult to assess the effects on MIC, as variation among the independent experiments was higher than that observed for the other antibiotics tested. The MIC of dicloxacillin was decreased by a factor of up to 4 in the presence of serum, but the results obtained for plasma seemed to be skewed due to heparin. At the same time, we detected an increase in the MBCs of rifampicin, teicoplanin, and tetracycline in human serum, but the true effect of plasma was difficult to determine due to interference caused by heparin.

**Figure 1:**
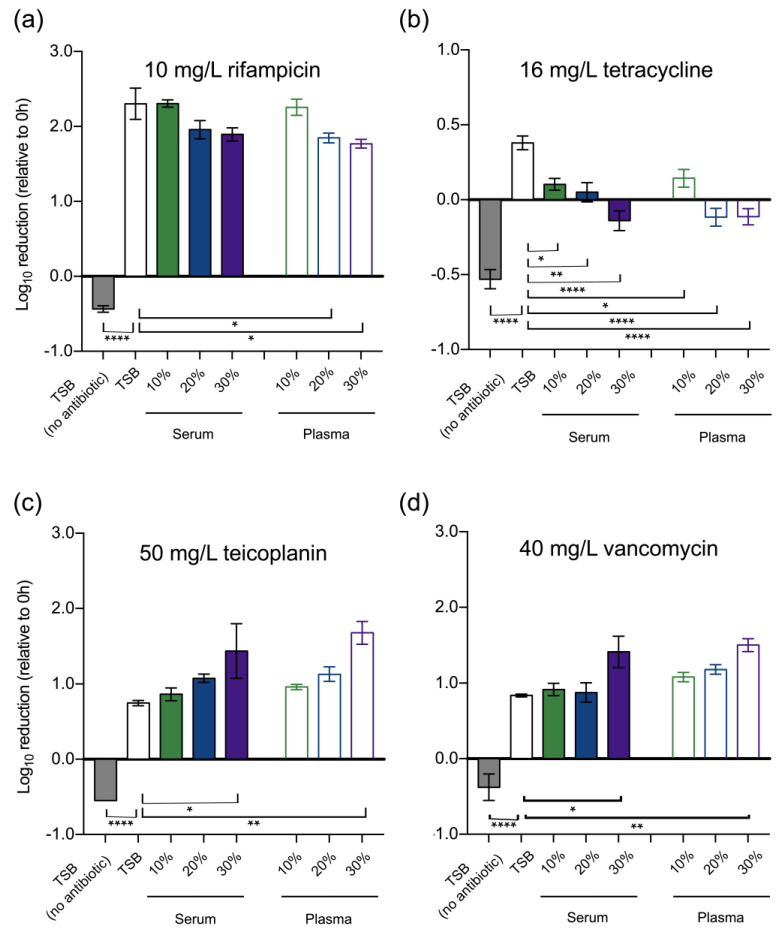
Human serum and plasma affect the activity of certain antibiotics against *S. epidermidis*. Reduction in the CFU counts of *S. epidermidis* RP62A grown in the presence of increasing concentrations of human serum and plasma upon 3 h of incubation with (**a**) rifampicin, (**b**) tetracycline, (**c**) teicoplanin, and (**d**) vancomycin at their respective PSCs. Data are represented as the mean ± SEM (*n* = 3, with technical duplicates). For some points, the error bars are shorter than the height of the symbol. Significant differences were determined by one-way ANOVA with Dunnett’s multiple comparisons tests. * *p* < 0.05; ** *p* < 0.01; **** *p* < 0.0001 (vs. TSB).

Although the MIC and MBC assays allowed us to estimate the general effect of human serum and plasma on antibiotic activity, we noted some inter-assay variability that hindered us from drawing more definite conclusions. The inaccuracy of this type of assay has been a matter of concern [24] and may explain the variations observed. To further gain knowledge into the actual effect of human serum and plasma, we then assessed antibiotic susceptibility by CFU counting after exposure to selected antibiotics at their PSC. Any confounding effects caused by heparin were discarded at this level (Figure S1). Concentrations of serum and plasma above 10% reduced the activity of rifampicin, with the effect being more pronounced in the presence of plasma (Figure 1a). A more noticeable effect was observed for tetracycline as antimicrobial activity was lost in the presence of higher concentrations of serum or plasma (Figure 1b). Interestingly, the opposite effect was observed for teicoplanin and vancomycin. Although the binding of teicoplanin and vancomycin to plasma proteins are fairly different (~95% vs. ~35%, respectively) [25,26], it was surprising to note that the presence of human serum or plasma significantly enhanced the activity of both antibiotics in a concentration-dependent manner (Figure 1c,d).

### 3.2. Effect of Host Serum Factors on Biofilm Formation

**Figure 2:**
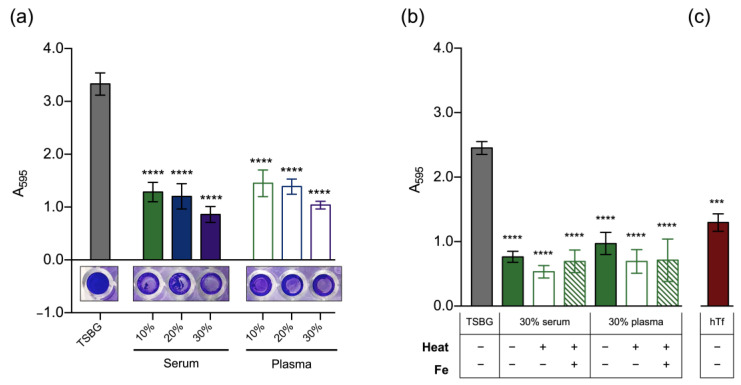
Biofilm formation by *S. epidermidis* is impaired in the presence of human serum and plasma. We assessed the effect of (**a**) increasing concentrations of human serum/plasma, (**b**) heat inactivation of serum/plasma with and without iron supplementation (Fe, 100 µM iron chloride), and (**c**) human transferrin (hTf, 1 g/L) on *S. epidermidis* biofilm formation ability. Bacteria were grown for 24 h at 37 °C on flat bottom 96-well microplates, and quantification of biofilm biomass was performed through crystal violet staining. Data are represented as the mean ± SEM (a: *n* = 5; b,c: *n* = 3, all with at least 6 technical replicates). Significant differences were determined by one-way ANOVA with Dunnett’s multiple comparisons tests. *** *p* < 0.001; **** *p* < 0.0001 vs. TSBG. TSBG, TSB supplemented with 0.4% glucose.

The persistent nature of *S. epidermidis* infections is generally attributed to the ability of this bacterium to grow as biofilms, which is thought to account for the reduced efficacy of antimicrobial therapies (reviewed in Büttner et al. [27]). Therefore, we assessed the ability of *S. epidermidis* to form biofilms in the presence of human serum or plasma as a means of simulating the conditions found in the host. Although biofilm formation still occurred under these conditions, the biomass level observed was significantly lower than that observed for the medium alone, even for the lowest concentration of serum/plasma tested (Figure 2a).

A possible inhibitory effect due to the presence of heparin in plasma was ruled out (Figure S2). To understand the nature of this effect, we assessed planktonic growth under the same conditions. While serum concentrations up to 20% had no observable effect, concentrations of 30% or higher led to a decreased growth rate, which resulted in a slightly lower optical density after 24 h. Nevertheless, no significant effect on the number of culturable cells was detected (Figure S3a,b). Regarding plasma, only the highest concentration tested (50%) significantly reduced both the optical density and CFU counts during the first 8 h of growth, an effect that was lost after 24 h (Figure S3c,d). Therefore, our findings indicate that any possible growth defect induced by serum and plasma had only a minor contribution to the anti-biofilm effect observed. At this stage, we hypothesized that such an effect is attributable to biofilm-specific mechanisms. We first assessed biofilm formation on microplates precoated with human serum/plasma, but we only found a small reduction in biomass (Figure S4a), indicating that mechanisms other than impaired adhesion capacity are in place. We then exposed 24 h-old biofilms to increasing concentrations of serum and plasma for an additional 24 h, but again, we found no significant reduction in biomass (Figure S4b). This is in line with previous results from our group [5] and indicates that the reduced biomass we detected was not the result of biofilm dispersal.

We also investigated the effect of heat inactivation of serum/plasma and found that it did not reverse biofilm inhibition (Figure 2b). Based on our previous findings on the importance of iron for *S. epidermidis* biofilms [19], we hypothesized that iron restriction may be a major factor behind the antibiofilm effect observed. Therefore, we assessed biofilm formation in the presence of iron-enriched serum/plasma and human transferrin. While the addition of iron did not restore biofilm formation (Figure 2b), we found that human transferrin at a concentration of 1 g/L (~30% of normal adult levels; reference range = 2–3 g/L [28]) produced a ~50% reduction in biofilm biomass (Figure 2c).

### 3.3. Gene Expression following Exposure to Host Serum Factors

**Figure 3:**
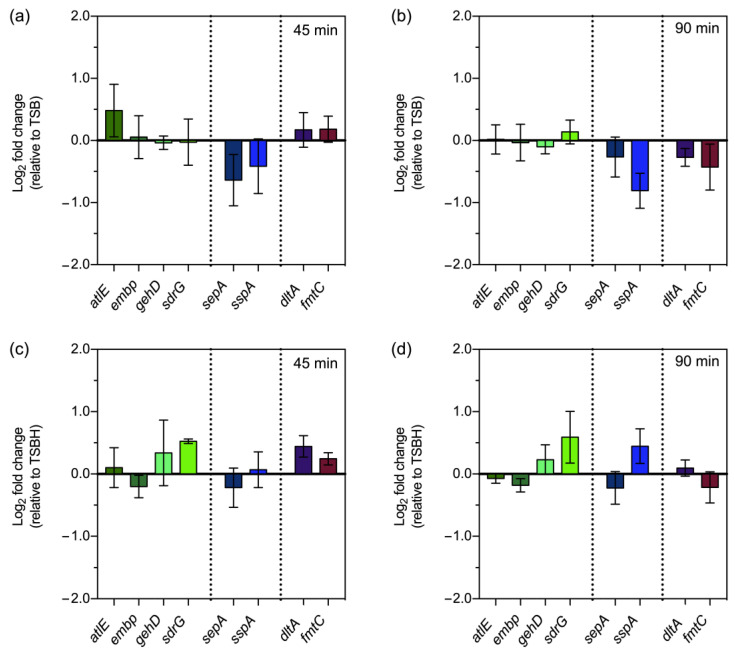
The presence of human serum and plasma induces alterations in the transcription of genes associated with key bacterial processes. *S. epidermidis* RP62A was grown for 45 and 90 min in TSB/TSBH (control conditions) or TSB containing 20% human serum (**a**,**b**) or plasma (**c**,**d**). Gene expression analysis was carried out by qPCR, where transcription levels were normalized to the expression of 16S rRNA and *gyrB*. The genes studied are involved in binding to extracellular matrix proteins and adhesion (*atlE*, *embp*, *gehD*, and *sdrG*), immune evasion (*sepA* and *sspA*), and bacterial membrane charge (*dltA* and *fmtC*). Fold change data were calculated according to the Pfaffl method and log-transformed (Log_2_). Data are represented as the mean ± SEM (*n* = 3, with technical duplicates in qPCR quantification). Log_2_ fold change values above and below 0 indicate the up- and downregulation of transcription, respectively, in comparison to the control conditions (TSB vs. serum; TSBH vs. plasma). TSBH, TSB supplemented with heparin.

To provide some mechanistic insights into the effects that we observed at the level of biofilm formation and antibiotic susceptibility, we investigated if exposure of planktonic bacteria to serum and plasma for short periods (45 and 90 min) induces relevant alterations in the expression of the selected genes. We quantified the transcription levels of genes associated with key bacterial processes, namely binding to host adhesive matrix molecules (*atlE*, *embp*, *gehD*, and *sdrG*), immune evasion (*sepA* and *sspA*), and bacterial surface charge (*dltA* and *fmtC*) (Figure 3). The most noteworthy finding was the change in the transcription of *dltA* and *fmtC*. After an initial upregulation, further exposure to serum or plasma resulted in the downregulation of these genes. This finding led us to hypothesize that host serum factors induce a stronger negative surface charge that renders *S. epidermidis* more susceptible to the activity of glycopeptides. Interestingly, we were not able to detect any significant change in the bacterial surface charge under our experimental conditions (Figure S5). The expression of genes mediating binding to host adhesive matrix molecules (*atlE*, *embp*, *gehD*, and *sdrG*) remained almost unchanged in the presence of serum, while exposure to plasma led to the upregulation of *sdrG*.

## 4. Discussion

A significant amount of our knowledge of *S. epidermidis* virulence traits is still derived from studies performed in vitro using standard laboratory culture media. While this approach has been fundamental for our understanding of multiple pathogenic mechanisms, it has been demonstrated that the presence of human factors may induce significant alterations in bacterial physiology [29,30].

The influence of molecules present in human blood, such as plasma proteins, in antibiotic susceptibility testing has long been recognized [31]. Although antibiotic susceptibility is routinely assessed in vitro using standard culture media, this does not represent the conditions found by the pathogens within the host, where the activity of antibiotics may be affected by a plethora of molecules present in human blood. Plasma protein binding has been generally accepted as an important factor accounting for reduced antimicrobial activity [31,32]. Although this study did not specifically address protein binding effects, the extent of the protein binding of rifampicin and tetracycline reported in the literature is relatively high (60–90% and 65%, respectively) [26]. Therefore, our findings demonstrate that the activity of rifampicin and tetracycline against *S. epidermidis* was impaired in the presence of human serum or plasma, most likely due to their high protein binding properties. We also found that the presence of human serum or plasma enhances the activity of glycopeptides. While the intrinsic antibacterial activity of serum/plasma may have partly led to reduced bacterial counts, the fact that it was only detectable for this class of antibiotics is a strong indicator that there are other mechanisms behind this effect. Enhanced vancomycin activity in the presence of human [33,34] and rat [35,36] sera against *S. aureus* and enterococci, respectively, has been reported. Conversely, Stratton and Weeks [37] observed the decreased activity of vancomycin against different staphylococcal and enterococcal isolates in the presence of human serum. However, this study used heat-inactivated serum, which may indicate that such an enhancing effect is driven by heat-labile molecules. Concerning teicoplanin, it has been reported that human serum either had no effect or mildly inhibited its activity against *S. aureus* [38,39]. Therefore, the enhancing effect we observed for teicoplanin may be species specific. Further studies with other *S. epidermidis* strains will help to elucidate this matter.

Interestingly, we also observed that the presence of human serum/plasma was very detrimental to biofilm formation. Recently, Skovdal et al. [40] reported that *S. epidermidis* biofilms formed in the presence of human plasma occur mostly as suspended aggregates and, most strikingly, major biofilm matrix components such as the polysaccharide intracellular adhesin (PIA) and the extracellular matrix-binding protein (Embp) are not required for biofilm formation in the presence of host factors. She et al. [10] found that human serum exhibits significant antibiofilm activity against the *S. epidermidis* RP62A strain and claimed that both heat-labile and heat-stable components account for that effect [10]. Our data indicate that the major serum inhibitory factor(s) is(are) heat-stable, suggesting that differences in the serum composition may account for slight disparities among studies. Moreover, our experiments suggest that transferrin-mediated iron sequestration is largely responsible for the detrimental effect of human serum/plasma on *S. epidermidis* biofilms. Further supporting this conclusion is the fact that: (i) transferrin was likely in its native state after the heat treatment since its thermal denaturation occurs only at temperatures above 60 °C [41], and (ii) some studies have reported the negative effect of human transferrin on bacterial adhesion and biofilm formation by staphylococci [10,42,43]. Collectively, there has been growing evidence reinforcing the concept that biofilm formation mechanisms in standard culture media may differ significantly from those that occur within the host. In the future, it would be worth identifying additional host serum factors responsible for the modulation of *S. epidermidis* biofilm formation that we and other authors have reported.

Lastly, our gene expression analysis revealed the combined downregulation of *dltA* and *fmtC* genes shortly after exposure to host serum factors. In *S. aureus*, it was demonstrated that the *dltABCD* operon is responsible for D-alanine incorporation into teichoic acids [44], and its disruption results in a stronger negative bacterial surface charge that leads to increased susceptibility to glycopeptide antibiotics [13] and an inability to colonize artificial surfaces, such as polystyrene and glass [45]. Similarly, Nishi et al. [12] demonstrated that mutations in *fmtC* lead to a more negative bacterial surface charge, although its effect on vancomycin susceptibility seems to vary according to the genetic background of the strain. In strains with a methicillin-resistant or vancomycin intermediate-resistant background, as is the case of *S. epidermidis* RP62A, *fmtC* inactivation seems to increase the susceptibility to vancomycin [12,46]. Although data from studies on *S. aureus* pointed out changes in the bacterial surface charge as the most reasonable mechanism behind the effect of host serum factors on antibiotic susceptibility and biofilm formation, our results do not support such a hypothesis. Regarding the expression of the remaining genes, the upregulation of *sdrG* in the presence of plasma was expected since SdrG mediates adhesion to fibrinogen [47], which is abundant in plasma but almost absent in serum. Sellman et al. [48] failed to detect SdrG on the bacterial surface of *S. epidermidis* cultured in TSB or human serum, although they found increased expression of SdrG during the early stages of infection (1 to 3 h) in a murine bacteremia model. Similarly, *sspA* was found to be upregulated in the presence of plasma but not serum. SspA, also known as GluSE [49] or Esp [50], is a serine protease that has been described as being able to degrade human fibrinogen [51,52]. Collectively, these results demonstrate that plasma-enriched growth conditions may be a good representation of the in vivo conditions that *S. epidermidis* finds during infection.

**Figure 4:**
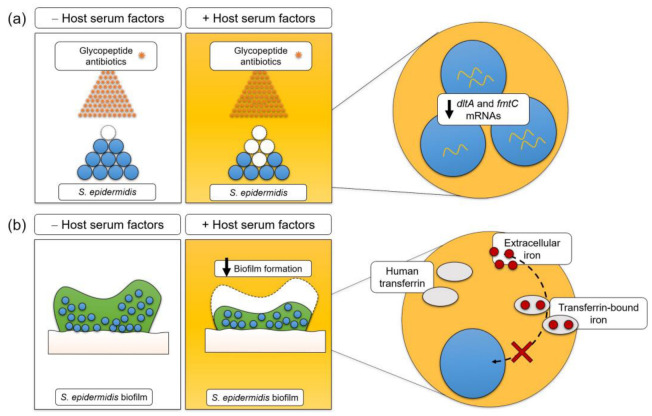
Illustration of the major findings regarding the influence of human factors present in plasma and/or serum on *S. epidermidis’s* susceptibility to glycopeptide antibiotics and the expression of genes associated with bacterial surface charge (*dltA* and *fmtC*) and biofilm formation. Our data demonstrate that human serum factors put a double burden on *S. epidermidis*: (**a**) not only do they lead to increased susceptibility to glycopeptide antibiotics, (**b**) but they also impair its ability to form biofilms on artificial surfaces, which we hypothesize is largely through a transferrin-mediated iron sequestration process.

In summary, we provide data that demonstrate that human serum factors put a double burden on *S. epidermidis*; not only do they lead to increased susceptibility to glycopeptide antibiotics, but they also impair its ability to form biofilms on artificial surfaces (Figure 4). The identification of the mechanisms behind this phenomenon may be pivotal in fostering the development of new antimicrobial strategies against this nosocomial pathogen. Additionally, this study stresses the need to introduce host factors into in vitro experiments to resemble the in vivo conditions and, in this way, increase the clinical relevance of the results obtained.

## Data Availability

The data presented in this study are available in the article and Supplementary Material.

## Supplementary Materials

The following supporting information can be downloaded at: https://www.mdpi.com/article/10.3390/pathogens12081064/s1. Figure S1: Effect of heparin on the activity of the antibiotics tested; Figure S2: Effect of heparin on *S. epidermidis* biofilm formation; Figure S3: Effect of human serum and plasma on *S. epidermidis* growth; Figure S4: Effect of human serum and plasma at different stages of biofilm formation; Figure S5: Effect of human serum on the bacterial surface charge; Table S1: List of primers used in this study; Table S2: List of antibiotics used in this study; Table S3: MIC ranges (in mg/L) of five antimicrobial agents for *S. epidermidis* RP62A in the presence of human serum and plasma. TSBH, TSB supplemented with heparin; Table S4: MBC ranges (in mg/L) of five antimicrobial agents for *S. epidermidis* RP62A in the presence of human serum and plasma. TSBH, TSB supplemented with heparin.
