# Supplementary material for: Host Soluble Factors Cause Changes in Staphylococcus epidermidis Antibiotic Susceptibility and Biofilm Formation Ability
Source: Pathogens. 2023 Aug 19;12(8):1064. doi: 10.3390/pathogens12081064 (PMC10459130; doi:10.3390/pathogens12081064)
Supplement: Supplementary file 1 [file pathogens-12-01064-s001.zip › pathogens-2522796-supplementary.pdf]

Supplementary data for

Host soluble factors cause changes in Staphylococcus epidermidis antibiotic susceptibility and biofilm formation ability

Fernando Oliveira [1], Vânia Gaio [1], Susana Brás1, Sofia Oliveira [1] and Angela França [1,2,*]

[1] Centre of Biological Engineering, LIBRO - Laboratory of Research in Biofilms Rosário Oliveira, University of Minho, Campus de Gualtar, 4710-057, Braga, Portugal
[2] LABBELS – Associate Laboratory, Guimarães/Braga, Portugal
* Correspondence: afranca@ceb.uminho.pt

Supplementary figures

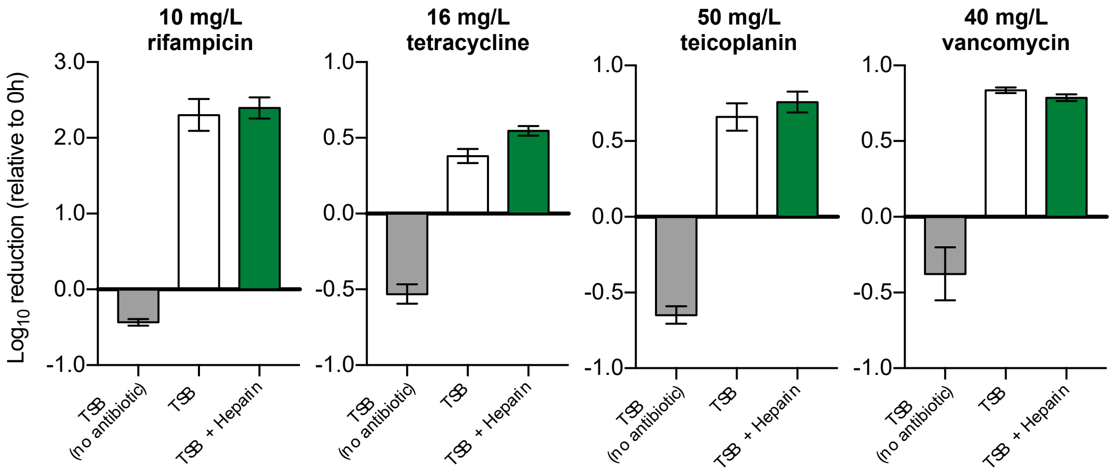

Figure S1. Effect of heparin on the activity of the antibiotics tested. Reduction in the CFU counts of S. epidermidis RP62A grown in the presence of heparin upon 3 hours of incubation with rifampicin, tetracycline, teicoplanin, and vancomycin at their respective peak serum concentration (PSC). Data are represented as mean ± SEM (n = 3-4). One-way ANOVA with Dunnett's multiple comparisons test found no differences between TSB and TSB plus heparin.

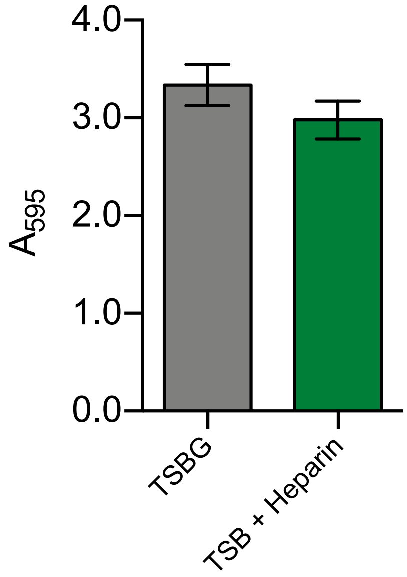

Figure S2. Effect of heparin on S. epidermidis biofilm formation. Data are represented as mean ± SEM (n = 5). One-way ANOVA with Dunnett's multiple comparisons test found no significant differences between TSB and TSB plus heparin.

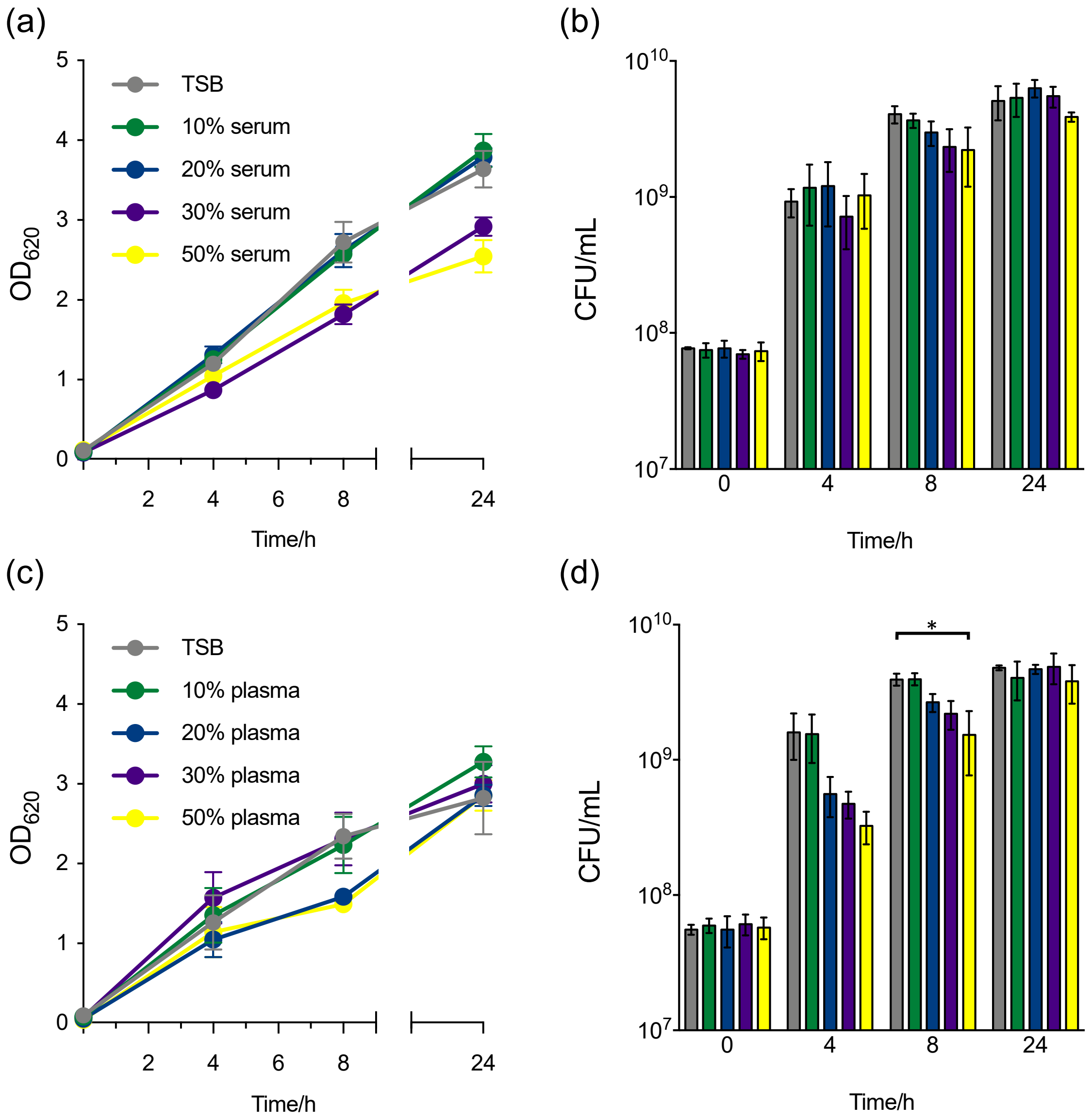

Figure S3. Effect of human serum and plasma on S. epidermidis growth. Bacteria were grown in conical flasks for 24 h at 37°C, 120 rpm in serum (a and b)- or plasma (c and d)- enriched TSB and growth was monitored regarding OD620 (a and c) and CFU counts (b and d). Data are represented as mean ± SEM (n = 3). Significant differences were determined by two-way ANOVA with Dunnett's multiple comparisons tests. *$P < 0.05$ vs TSB.

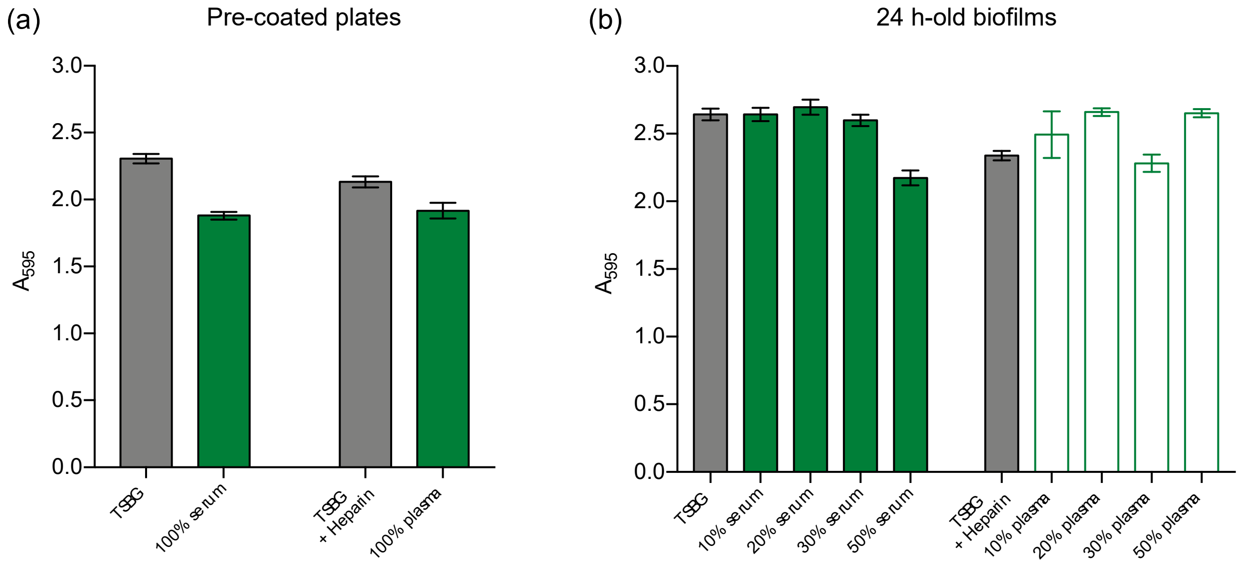

Figure S4. Effect of human serum and plasma at different stages of biofilm formation. (a) Biofilm formation on microplates precoated with human serum/ plasma; (b) Effect of human serum/plasma on 24 h-old biofilms. Data represent the mean ± SEM of 6 technical replicates from a single experiment.

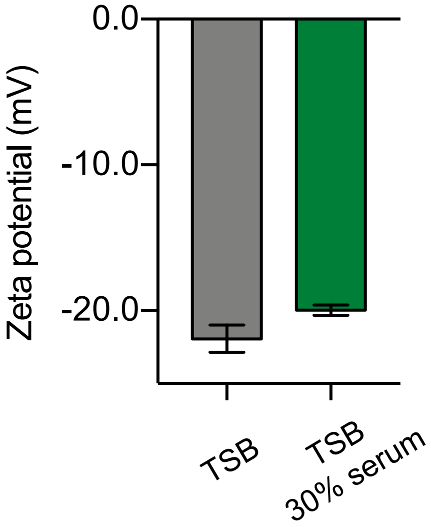

Figure S5. Effect of human serum on the bacterial surface charge. Data represent the mean ± SEM of 10 zeta potential measurements from a single experiment.

Supplementary tables

Table S1. List of primers used in this study.

**Target gene**	**Forward sequence (5′- 3′)**	**Reverse sequence (5′- 3′)**	**Product size (bp)**	**Efficiency (%)**
16S	GGGCTACACACGTGCTACAA	GTACAAGACCCGGGAACGTA	176	91.0
gyrB	GCATTTGGTACGGGTATTGG	CATCAACATCGGCATCAGTC	88	101.5
atlE	GTAGATGTTGTGCCCCAAGG	ACCTGATAGCGCTGAAAACG	201	98.5
embp	ATCGCAGCAAGTACATCCACCA	ATATTCTGTTGCATCCGCACGA	112	102.9
gehD	AATCGAGCGCTTTAGTTCC	TGGTGGTACGGTATCCGATT	176	103.1
sdrG	GGACGATTACACGACGATACAGCA	GAGGCAAGTCACCTTGTCCTTGA	140	98.0
sepA	TCTTAAGGCATCTCCGCCTA	GTCTGGTGCGAATGATGTTG	196	89.0
sspA	GTGGCTAATGGTTTGTCACC	TCCTAATGGCAAATTTGTGG	201	105.1
dltA	ACACATATGGACCAACTGAAGCTA	CCTCAATGACAAGTTCTCCTTCTT	150	94.0
fmtC	CGCCCTCATCATAGCATTG	CCAATTGGATCACCCAAAAC	182	103.8

Table S2. List of antibiotics used in this study

**Antimicrobial agent**	**Class**	**Mechanism of action**
Dicloxacillin	β-lactams	Inhibition of cell wall synthesis
Rifampicin	Rifamycins	Inhibition of RNA synthesis
Teicoplanin	Glycopeptides	Inhibition of cell wall synthesis
Tetracycline	Tetracyclines	Inhibition of protein synthesis
Vancomycin	Glycopeptides	Inhibition of cell wall synthesis

Table S3. MIC ranges (in mg/L) of five antimicrobial agents for *S. epidermidis* RP62A in the presence of human serum and plasma. TSBH, TSB supplemented with heparin (*n* = 3-4)

Antimicrobial agent	TSB	Human serum			TSBH	Human plasma		
		10%	20%	30%		10%	20%	30%
Dicloxacillin	4-8	4	2-4	2-4	2	2	2	2-4
Rifampicin	0.004	0.008	0.008-0.016	0.008-0.031	0.002-0.004	0.008	0.008	0.008-0.016
Teicoplanin	1-4	2-4	2-4	2-4	1-2	2-4	2-4	4
Tetracycline	0.25	0.25	0.25	0.125-0.25	0.25	0.25	0.25	0.25-0.5
Vancomycin	2-4	2	2-4	2	2	2	2	2

Table S4. MBC ranges (in mg/L) of five antimicrobial agents for *S. epidermidis* RP62A in the presence of human serum and plasma. TSBH, TSB supplemented with heparin (*n* = 3-4)

**Antimicrobial agent**	**TSB**	**Human serum**			**TSBH**	**Human plasma**		
		**10%**	**20%**	**30%**		**10%**	**20%**	**30%**
Dicloxacillin	≥64	≥64	>64	>64	>2	>64	>64	>64
Rifampicin	0.16	0.031-0.063	0.031	0.031-0.063	0.008	0.031-0.063	0.031-0.063	0.031 - ≥ 0.063
Teicoplanin	4	≥8	≥8	≥8	4 - >8	>8	≥8	≥8
Tetracycline	0.5-4	>4	>4	>4	>4	>4	>4	>4
Vancomycin	>8	≥8	>8	≥8	8	4 - >8	4 - >8	4 - ≥8
